# Abnormal Brain Oscillations in Developmental Disorders: Application of Resting State EEG and MEG in Autism Spectrum Disorder and Fragile X Syndrome

**DOI:** 10.3389/fnimg.2022.903191

**Published:** 2022-05-27

**Authors:** Sophia Liang, Maria Mody

**Affiliations:** ^1^College of Arts and Sciences, Harvard University, Cambridge, MA, United States; ^2^Athinoula A. Martinos Center for Biomedical Imaging, Massachusetts General Hospital and Harvard Medical School, Charlestown, MA, United States

**Keywords:** Autism Spectrum Disorder (ASD), Fragile X Syndrome (FXS), electroencephalography (EEG), magnetoencephalography (MEG), resting state, brain oscillations

## Abstract

Autism Spectrum Disorder (ASD) and Fragile X Syndrome (FXS) are neurodevelopmental disorders with similar clinical and behavior symptoms and partially overlapping and yet distinct neurobiological origins. It is therefore important to distinguish these disorders from each other as well as from typical development. Examining disruptions in functional connectivity often characteristic of neurodevelopment disorders may be one approach to doing so. This review focuses on EEG and MEG studies of resting state in ASD and FXS, a neuroimaging paradigm frequently used with difficult-to-test populations. It compares the brain regions and frequency bands that appear to be impacted, either in power or connectivity, in each disorder; as well as how these abnormalities may result in the observed symptoms. It argues that the findings in these studies are inconsistent and do not fit neatly into existing models of ASD and FXS, then highlights the gaps in the literature and recommends future avenues of inquiry.

## Introduction

Autism Spectrum Disorder (ASD) and Fragile X Syndrome (FXS) are neurodevelopmental disorders with known comorbidity and share many features.

The symptoms of both disorders include repetitive motions, sensory hypersensitivity, echolalia, attention deficits, anxiety, and impaired social interaction such as poor eye contact, perseveration in speech, and aggression (Belser and Sudhalter, 2001; Charman, 2003; Belmonte and Bourgeron, 2006; Poole et al., 2018; Chernenok et al., 2019). Both disorders occur more often in males than females, with a 3:1 bias in ASD (Loomes et al., 2017) and a 2:1 bias in FXS (Hunter et al., 2014). The estimated prevalence of ASD in FXS ranges from 5 to 60 percent (Belmonte and Bourgeron, 2006), and FXS is the leading monogenic cause of ASD, accounting for around 5 percent of cases (Simberlund and Veenstra-VanderWeele, 2018).

However, there are crucial differences between the two disorders. FXS is caused by a mutation of the fragile X mental retardation type 1 (*FMR1*) gene that blocks its transcription. ASD, on the other hand, is an entirely behavioral diagnosis with polygenetic, epigenetic, and environmental roots (Persico and Bourgeron, 2006). Its two domains of diagnostic criteria in the DSM-5 are a) deficits in social communication and interaction and b) restricted, repetitive behaviors (Yaylaci and Miral, 2017). The two disorders show some subtle differences in their characteristic symptoms — for example, McDuffie et al. (2015) found that individuals with FXS displayed less impaired social smiling and more stereotyped motor behaviors than those with idiopathic ASD in a severity-matched analysis. Brain differences have also been observed in structural magnetic resonance imaging. Hoeft et al. (2011) found that, compared to typically developing controls (TD), the frontal and temporal areas involved in social cognition are larger in idiopathic ASD but smaller in FXS.

ASD and FXS have been described as disorders of connectivity (Rippon et al., 2007; Haberl et al., 2015). Structural connectivity, or the physical connections of synapses and tracts, appears to be impaired with fewer connections, for example, between the amygdala and other brain regions in both disorders. However, the brain's functional connectivity, or the temporal correlations between the activity of spatially distinct regions, is also disrupted in ASD and FXS (Hull et al., 2017).

This review examines functional connectivity through the lens of electroencephalography (EEG), quantitative electroencephalography (qEEG), and magnetoencephalography (MEG) recording techniques. Examining the large-scale organization of the brain in ASD and FXS can lend insight into the biomarkers and etiology of these disorders, for improved diagnosis and treatment.

Compared to functional magnetic resonance imaging (fMRI), EEG is less expensive, more portable, and offers higher temporal resolution at the cost of some spatial resolution. EEG data can be interpreted using spectral band analysis, whereby the signal is decomposed into frequency bands: delta (1-3 Hz), theta (4-7 Hz), alpha (8-12 Hz), beta (13-30 Hz), and gamma (31-50 Hz). These bands are believed to be functionally distinct, though the upper and lower boundary frequencies that define each varies considerably in the literature (Newson and Thiagarajan, 2019). EEG “power” represents the amount of activity in a given frequency band of the signal (Nunez and Srinivasan, 2005). Functional connectivity is quantified using a variety of metrics including coherence, synchronization likelihood, phase lag index, and phase-amplitude coupling. EEG and MEG signals originate from the same neural sources and have high temporal resolution, but MEG is less affected by tissue properties. MEG is more sensitive to currents that are tangential to the surface of the scalp, whereas EEG is sensitive to both tangential and radial currents. MEG is more expensive and unportable (Singh, 2014), though there appear to be exciting new developments in the field of wearable optically pumped magnetometers (Boto et al., 2018).

We focus on resting-state studies conducted when participants are not given any external stimuli nor instructed to engage in any particular task. Data collection is straightforward and at lower risk of being confounded by cognitive or motor impairments; the brain is spontaneously active even in this “resting” state, reflecting patterns similar to those generated under active task conditions.

Building on the work of Devitt et al. (2015), we aim to compare resting-state EEG and MEG studies of ASD and FXS, to more thoroughly break down the frequency bands and brain areas implicated in each disorder and examine how these abnormalities in functional connectivity may contribute to the observed symptoms.

## Resting State EEG and MEG in ASD and FXS

The tables in this section synthesize the findings of EEG and MEG resting state studies in ASD and FXS. The “higher” or “lower” results refer to the direction of the difference, in either power or functional connectivity, observed in ASD (Table 1) or FXS (Table 2) compared to typically developing controls. The results are specific to brain regions (row) and frequency bands (column), with the exception of “global” results, where differences were noted throughout the entire brain.

**Table 1:** Power and connectivity abnormalities implicated within and across brain areas in ASD.

	**Delta**	**Theta**	**Alpha**	**Beta**	**Gamma**
Global	• Higher power (1, 5, 23) • Lower intrahemispheric (9, 17, 20) • Higher intrahemispheric (23) • Higher **interhemispheric** (23)	• Higher power (6, 14) • Lower power (23) • Higher intrahemispheric (6) • Lower intrahemispheric (9, 17, 20, 21)	• Higher power (14) • Lower power (1, 5, 6, 20, 23) • Higher intrahemispheric (23) • Lower intrahemispheric (20, 21) • Higher **interhemispheric** (1)	• Higher power (6, 23) • Lower power (20) • Higher intrahemispheric (23) • Lower intrahemispheric (17, 21) • Higher **interhemispheric** (1, 23)	• Higher power (23) • Higher intrahemispheric (22, 23) • Lower intrahemispheric (21) • Higher **interhemispheric** (22, 23)
Frontal	• Higher power (11, 14, 20) • Lower power (2, 9) • Higher connectivity within region (12, 21) • Lower connectivity with occipital (12) • Lower **interhemispheric** (9) • Lower connectivity with parietal (19)	• Higher (3, 11, 20) • Lower power (2) • Higher connectivity within region (6) • Lower **interhemispheric** (9)	• Higher power (8) • Lower power (2) • Lower connectivity within region (6) • Lower connectivity with all other brain regions (6) • Higher connectivity with occipital (22) • Lower **interhemispheric** (13, 18)	• Lower connectivity within region (15, 22) • Lower connectivity with occipital (19)	• Higher power (10) • Higher connectivity with temporal (16, 19) • Higher connectivity within region (19)
Temporal	• Lower power (2) • Lower connectivity within region (8, 19) • Higher **interhemispheric** (20) • Lower **interhemispheric** (9)	• Higher power (9) • Lower power (2) • Higher connectivity within region (6) • Lower connectivity within region (8) • Lower **interhemispheric** (9)	• Higher power (14) • Lower power (2) • Lower connectivity with frontal (6) • Higher connectivity with occipital (22) • Higher connectivity with other regions (21) • Lower connectivity with other regions (13) • Lower **interhemispheric** (9, 13, 18)	• Lower connectivity within region (15, 22) • Higher connectivity within region (19)	• Higher power (14) • Lower power (24) • Higher connectivity with frontal, parietal, occipital (16) • Higher connectivity with frontal (19) • Higher connectivity within region (19)
Parietal	• Lower **interhemispheric** (9) • Lower connectivity with frontal (19) • Lower connectivity within region (19)	• Higher power (9, 14) • Lower power (2) • Lower **interhemispheric** (9, 20) • Lower connectivity within region (19) • Lower connectivity with other brain regions (19)	• Higher power (4, 14) • Lower connectivity with frontal (6) • Higher connectivity with occipital (22) • Higher connectivity with other regions (21) • Lower **interhemispheric** (18) • Lower connectivity within region (19) • Lower connectivity with other brain regions (19)	• Higher power (7, 14) • Lower **interhemispheric** (9) • Lower connectivity with occipital (19)	• Higher power (7, 14) • Higher connectivity with temporal (16)
Occipital	• Lower connectivity with frontal (12) • Lower **interhemispheric** (9)	• Higher power (9, 14) • Lower **interhemispheric** (9, 20) • Lower connectivity within region (19) • Lower connectivity with other brain regions (19)	• Higher power (14) • Lower connectivity with frontal (6) • Higher connectivity with all other brain regions (21, 22) • Lower connectivity with other brain regions (19) • Lower connectivity within region (19)	• Higher power (14) • Lower **interhemispheric** (9) • Lower connectivity within region (19) • Lower connectivity with frontal (19) • Lower connectivity with parietal (19)	• Higher power (14, 25) • Higher connectivity with temporal (16)

*Brain regions have been color-coded to make the connections between them more apparent; interhemispheric connectivity between the same lobe in different hemispheres (e.g., the left frontal lobe with the right frontal lobe) have been bolded*.

*Key: 1. Cantor et al. (1986); 2. Dawson et al. (1995); 3. Daoust et al. (2004); 4. Sutton et al. (2005); 5. Chan et al. (2007); 6. Murias et al. (2007); 7. Orekhova et al. (2007); 8. Stroganova et al. (2007); 9. Coben et al. (2008); 10. Sheikhani et al. (2009); 11. Pop-Jordanova et al. (2010); 12. Barttfeld et al. (2011); 13. Tsiaras et al. (2011); 14. Cornew et al. (2012); 15. Duffy and Als (2012); 16. Sheikhani et al. (2012); 17. Barttfeld et al. (2013); 18. Carson et al. (2014); 19. Ye et al. (2014); 20. Elhabashy et al. (2015); 21. Ghanbari et al. (2015); 22. Kitzbichler et al. (2015); 23. Machado et al. (2015); 24. Maxwell et al. (2015); 25. Vandewouw et al. (2021)*.

**Table 2:** Power and connectivity abnormalities implicated within and across brain areas in FXS.

	**Theta**	**Alpha**	**Beta**	**Gamma**
Global	• Higher power (A)	• Lower power (A, B) • Lower long-range connectivity (C) • Lower connectivity (B)	• Lower long-range connectivity (C) • Lower connectivity (B)	• Higher short-range connectivity (C)
Frontal	• Higher power (C) • Higher connectivity with parietal and occipital (B)	• Lower short-range connectivity (B) • Lower connectivity with parietal and occipital (B)	• Lower short-range connectivity (B) • Lower connectivity with parietal and occipital (B)	• Higher power (C)
Parietal	• Higher connectivity with frontal and occipital (B)	• Lower connectivity within region (B) • Lower connectivity with frontal (B)	• Lower connectivity with frontal (B)	
Occipital	• Higher power (C) • Higher connectivity with frontal and parietal (B)	• Lower connectivity within region (B) • Lower connectivity with frontal (B)	• Lower connectivity with frontal (B)	• Higher power (C)

*Key: A. Van der Molen and Van der Molen (2013); B. van der Molen et al. (2014); C. Wang et al. (2017). Brain regions have been color-coded to make the connections between them more apparent; interhemispheric connectivity between the same lobe in different hemispheres have been bolded*.

As shown in Tables 1, 2, power abnormalities, overconnectivity, and underconnectivity across frequency bands and brain regions are implicated in ASD and FXS. Yet these differences are far from consistent in the literature and do not appear to fall neatly into one model (e.g., the “U-shaped profile” of ASD to describe excessive power in low-frequency and high-frequency bands) (Wang et al., 2013). Only significant differences are reported in the tables, but many of the studies found no differences between the ASD/FXS and control groups for a given frequency band and brain area. It is particularly difficult to draw conclusions from the FXS data, as this review identified only three resting state studies in FXS. The following section will discuss some of the general patterns revealed in the literature, how these electrophysiological abnormalities may relate to ASD and/or FXS symptoms, possible reasons behind the (many) inconsistencies, and future avenues for research.

### Delta

Delta power is elevated globally in ASD (and insufficiently studied in FXS). Enhanced delta power is commonly observed among low-functioning children with ASD in studies that involve doing a task (Wang et al., 2013), as well as children with learning disabilities (Fonseca et al., 2006) and those born pre-term (Rommel et al., 2017). The delta band plays roles ranging from sustained attention to decision making to motivation, and it has been proposed that increased resting delta power is a general marker of brain trauma, pathology, or neurotransmitter disturbances (Başar-Eroglu et al., 1992; Kirmizi-Alsan et al., 2006; Knyazev, 2012; Rommel et al., 2017).

In ASD, delta connectivity appears to be increased within the frontal lobe but decreased elsewhere. As slower oscillations are usually associated with longer range connections, this could reflect a failure of top-down synchronization and poorer inhibitory regulation. It is also reflective of hyperconnectivity seen within the frontal region more generally.

### Theta

Theta power is elevated globally in both ASD and FXS. The association of the theta band with response inhibition, focused attention, and working memory, as well as its negative correlation with default mode network activity, could explain the cognitive and attention deficits observed in these disorders (Başar-Eroglu et al., 1992; Klimesch et al., 2005; Kirmizi-Alsan et al., 2006; Scheeringa et al., 2008). It is consistent with findings of higher theta power in other psychiatric disorders including ADHD, schizophrenia, and learning disabilities (Clarke et al., 1998; Fonseca et al., 2006; Newson and Thiagarajan, 2019). Neurofeedback training aimed at reducing theta overactivity in patients with ASD results in long-lasting improvements in social and executive function, perhaps mediated by increased flexibility of the DMN (Kouijzer et al., 2009).

Individuals with FXS have higher connectivity in the theta band compared to control subjects, and individuals with ASD have increased theta connectivity in the frontal and temporal lobe but decreased theta connectivity in other areas. Theta oscillations have been linked to the top-down processing of internal mental context (von Stein and Sarnthein, 2000) and glutamatergic circuit activity (Gallinat et al., 2006), so an excess of such activity in the higher-order brain regions may explain the behavioral disinhibition seen in these disorders, while a deficit in theta connectivity in the sensory brain regions may explain the sensory hypersensitivity, or lack of inhibition.

### Alpha

Wang et al. (2017) found that alpha power was diminished in individuals with FXS; this decrease was correlated with greater social impairment and hypersensitivity to sensory stimuli observed clinically. These results are consistent with studies showing that the alpha band is involved in inhibitory control and correlated with lower arousal levels (Klimesch, 1996; Barry et al., 2004; Klimesch et al., 2007). Alpha oscillations may reflect a mechanism to suppress sensory information during selective attention (Foxe and Snyder, 2011). The data on alpha power in ASD are mixed. The U-shaped profile of power, whereby alpha power is reduced in individuals with ASD, is a popular model in the literature (Wang et al., 2013). However, several studies found an excess of alpha power instead. This, too, might be a compensatory mechanism similar to that proposed for beta, insofar as alpha power appears to increase for tasks demanding greater attentional control (Benedek et al., 2014; Mathewson et al., 2015). Furthermore, elevated alpha power is associated with greater autistic trait expression in the non-clinical general population. Moore and Franz (2017) found that increased relative alpha power in typically developing adults is associated with increased aloofness measured by the Broad Autism Phenotype Questionnaire (Moore and Franz, 2017); similarly, Carter Leno et al. (2018) found that among typically developing adults with subthreshold ASD trait expression, elevated resting-state alpha power was significantly correlated with behavioral rigidity in ASD. The suppression of alpha activity is an indicator of mirror neuron system activity, which is required for imitating behavior (Bernier et al., 2007). The elevated alpha power seen in ASD could well be linked to mirror neuron system dysfunction in ASD and the resulting social impairments.

### Beta

Beta waves are associated with alertness, motor behavior, and the direction of attention (Neuper and Pfurtscheller, 2001; Güntekin et al., 2013). ADHD is characterized by reduced beta power (Newson and Thiagarajan, 2019), yet paradoxically, the attention deficits observed in ASD are coupled with an elevation in beta power. This may be a compensatory mechanism for the social deficits also seen in ASD — Palacios-García et al. (2021) found that psychosocial stress can evoke higher beta power, perhaps as a top-down modulator to redirect attention to the stressful task at hand. Beta connectivity, on the other hand, is generally lower in ASD as well as FXS. van der Molen et al. (2014) suggest this is an indicator of immature cortical networks, since over the course of typical development, low-frequency synchronization decreases and high-frequency synchronization increases. Ye et al. (2014) found that individuals with ASD showed reductions in beta synchronization during a face processing task, suggesting a role for this frequency in social-emotional processes in ASD.

### Gamma

Wang et al. (2017) found that gamma power is elevated in FXS. Increased gamma power is correlated with social communication abnormalities, auditory hypersensitivity, and reductions in neurocognitive abilities in FXS (Ethridge et al., 2017, 2019; Wang et al., 2017). Orekhova et al. (2007) found that gamma power is positively correlated with degree of developmental delay in boys with ASD. However, Maxwell et al. (2015) found decreased gamma power among individuals with ASD compared to controls, and lower power was correlated with increased autism severity as measured by the Social Responsiveness Scale. Wilkinson et al. (2019) found that gamma power was lower in high-risk toddlers without ASD than in low-risk toddlers. Yet, among the high-risk group, reduced gamma was associated with improved language ability regardless of later ASD diagnosis. It is thus unclear whether lower gamma power is directly associated with cognitive deficits or is a compensatory mechanism for other processes that raise gamma power. The answer will likely vary between groups (ASD, high-risk, low-risk) and depend on sex as well as stage of development, so further research is needed to elucidate the role of the gamma band in ASD.

Gamma connectivity, unlike power, is generally increased across all brain regions in ASD. This broad difference is consistent with gamma's posited function as an elemental part of cortical computation, serving to segment and select between inputs (Fries, 2009). More specifically, Ye et al. (2014) found that the inferior frontal gyrus, orbitofrontal areas, amygdalae, and superior temporal gyrus, which are implicated in social cognition, were hyperconnected in the gamma band. Atypical connectivity could disrupt the interactions between these regions and other parts of the brain and lead to the socioemotional deficits seen in ASD. The frontal and temporal lobes appear to be the most heavily affected brain regions in ASD, with a general pattern of underconnectivity between these lobes and all other areas. Courchesne and Pierce (2005) hypothesize that these brain regions, responsible for higher-order cognitive and social functions, are later to mature and form synapses with a far greater number of neurons compared to the posterior cortices. Thus, their disproportionate disruption in ASD is consistent with the intact early development, followed by progressively greater abnormalities in the next few years, that is observed in the disorder. Specifically in the gamma band, though, individuals with ASD exhibit *higher* connectivity between the temporal lobe and other brain areas, which may account for the atypical language skills and memory difficulty seen in ASD. Interestingly, while frontal lobe connectivity is similarly affected in FXS and ASD, there appears to be little disruption in the temporal lobe in FXS (An et al., 2018).

### Interhemispheric Connectivity

Previous reviews in this field have largely neglected to discuss disrupted connectivity between hemispheres observed in ASD or FXS, but such disruptions have been frequently reported. Cantor et al. (1986) proposed that the higher interhemispheric connectivity they measured in individuals with ASD was an indicator of lack of cerebral differentiation, which has been linked to lower cognitive capabilities. However, most other ASD studies found a *decrease* in interhemispheric connectivity, consistent with a decrease in the volume of the corpus callosum in some autism subtypes (Alexander et al., 2007). Interhemispheric underconnectivity may explain why lateralized speech and social communication functions are disrupted in ASD. It could also explain intellectual deficits, since it may be more efficient for two hemispheres to interact while processing information than for either one to do it alone (Belger and Banich, 1992). None of the FXS studies found differences in interhemispheric connectivity. Further research on abnormal interhemispheric connectivity in these disorders — the direction of the change and the potential causes — is warranted.

## Limitations and Future Directions

The often-contradictory findings reported may arise from the variety of experimental methods used. Studies differed in the definition of each frequency band, the age of the subjects and the metrics used to quantify functional connectivity. Though they all used resting-state paradigms, some involved eyes-open conditions, while others were done with eyes closed. The EEG and MEG recordings could vary in their accuracy and precision based on the number of sensors used, and their distance from the participant's head (the MEG helmet is one size fits all), subject movement during recording, and how the data were analyzed to map the source space to the signal space and filter artifacts. Not all of the studies on ASD explicitly excluded participants with comorbid FXS, nor vice versa, Furthermore, terms such as “local,” “long-distance,” and “short-range” connectivity appear frequently in the literature but are defined only ambiguously, if at all.

The literature reflects the spatial resolution limitations of EEG and MEG; few of the power or connectivity differences are reported in greater specificity than the general cortical lobe that is implicated. Future research should capture more spatially specific sources, as potential nodes in a connectivity matrix. In the context of this paper thus far, “resting state” has been used as an experimental paradigm. However, the last decade has witnessed increasing attention being paid to resting state *networks* (RSNs), such as the default mode network and the dorsal attention network, as a feature of the brain. These networks are comprised of spatially distinct regions that are functionally connected when the brain is at rest. While there is not a one-to-one correspondence between RSNs and frequency bands, particular bands do appear to be implicated in each network; for example, gamma is elevated in the DMN at rest (Nair et al., 2018). Multimodal imaging combining EEG, MEG and fMRI could be used to integrate the spatial and temporal markers of these disorders.

The inconsistencies may also reflect the inherent heterogeneity of these disorders, especially ASD. Future research may need to break down the ASD label into behavioral and/or genetic subtypes as well as take developmental changes into account. There remains a general dearth of research on neural oscillations in FXS — which, given its more straightforward nature as a monogenic disorder and the considerable overlap between the two disorders, may be an overlooked pathway to understanding many features of ASD.

In conclusion, the EEG and MEG studies reveal interesting, if inconsistent, patterns in power and connectivity disruptions that hint at mechanisms underlying the symptoms in ASD and FXS. A more standardized analysis approach could help hone RS measures for use in targeted interventions.

## Author Contributions

MM conceived and designed the outline of this manuscript. SL prepared the manuscript assisted by MM. Both authors contributed equally to critically reviewing this work and approved the final version of the paper.

## Funding

This work was an outcome of research funded by the Nancy Lurie Marks Family Foundation (PI: MM) and the Office of the Assistant Secretary of Defense for Health Affairs, through the Peer Reviewed Medical Research Program under Award No. W81XWH-17-1-0228 (PI: Brownell and MM) in the areas of ASD and FXS.

## Conflict of Interest

The authors declare that the research was conducted in the absence of any commercial or financial relationships that could be construed as a potential conflict of interest.

## Publisher's Note

All claims expressed in this article are solely those of the authors and do not necessarily represent those of their affiliated organizations, or those of the publisher, the editors and the reviewers. Any product that may be evaluated in this article, or claim that may be made by its manufacturer, is not guaranteed or endorsed by the publisher.
